# Comparison of systemic immune-inflammation index (SII), early warning score (ANDC) and prognostic nutritional index (PNI) in hospitalized patients with malignancy, and their influence on mortality from COVID-19

**DOI:** 10.1186/s13027-021-00400-4

**Published:** 2021-09-15

**Authors:** Muge Bilge, Isil Kibar Akilli, Ekrem Bilal Karaayvaz, Aylia Yesilova, Kadriye Kart Yasar

**Affiliations:** 1 Department of Internal Medicine, Prof. Dr. Cemil Tascioglu City Hospital, University of Health Sciences, Darulaceze Street, No: 27 Sisli, 34384 Istanbul, Turkey; 2 Department of Pulmonary Disease, Sisli Hamidiye Etfal Training and Research Hospital, University of Health Sciences, Halaskargazi Street, 34371 Istanbul, Turkey; 3 Department of Cardiology, Istanbul Medical Faculty, University of Istanbul, Turgut Ozal Millet Street, Fatih, 34093 Istanbul, Turkey; 4 Department of Infectious Disease, Bakirkoy Dr. Sadi Konuk Training and Research Hospital, University of Health Sciences, Dr. Tevfik Saglam Street, No: 11, Bakirkoy, 34147 Istanbul, Turkey

**Keywords:** COVID-19 pneumonia, Malignancy, SII, PNI, ANDC

## Abstract

**Introduction:**

We evaluated several biological indicators based on inflammation and/or nutritional status, such as systemic immune-inflammation index (SII), early warning score (ANDC) and prognostic nutritional index (PNI) in hospitalized COVID-19 patients with and without malignancies for a prognostic significance.

**Methodology:**

This is a retrospective and observational study on 186 patients with SARS-CoV-2, who were diagnosed with COVID-19 by real-time PCR testing and hospitalized due to COVID-19 pneumonia. 75 patients had various malignancies, and the rest (111), having a similar age and comorbidity profile based on propensity score matching, had no malignancy.

**Results:**

None of the measures as neutrophil to lymphocyte ratio, platelet to lymphocyte ratio, monocyte to lymphocyte ratio, SII, PNI or ANDC was found to be significantly different between two groups. Odds ratio for the mortality, OR 2.39 (%95 CI 1.80–3.16) was found to be significantly higher for the malignancy group, even though the duration of hospitalization was statistically similar for both groups. PNI was found to be significantly lower for deceased patients compared with survivors in the malignancy group. Contrarily, ANDC was found to be significantly higher for deceased patients in the malignancy group.

**Conclusions:**

PNI and ANDC have independent predictive power on determining the in-hospital death in COVID-19 malignancy cases. It is suggested that ANDC seems to be a more sensitive score than SII in COVID-19 cases with malignancies.

## Introduction

The novel coronavirus SARS-CoV-2 appears in various clinical forms. Rapidly progressing hypoxemia and acute respiratory distress syndrome are commonly observed in patients with severe acute respiratory syndrome due to SARS-CoV-2, viral pneumonia and/or cytokine storm leading to a consequent hyperinflammatory state [1]. Cancer disease can make the patients more susceptible to the viral infections of COVID-19, as patients with cancer show immunocompromised health conditions [2]. However, the infection can rapidly progress to acute respiratory failure, sepsis, and eventually to death, especially in those patients who are susceptible to cancer and to poor nutritional status [3].

It was reported that the prognostic nutritional index (PNI), calculated by combining serum albumin levels and the total circulating lymphocyte count, could reflect the nutritional and immunological status of patients having cancer [4]. Previous studies have shown that lower PNI was related to a lower survival in cancer cases of pretreatment or post-surgery [5–7]. Also, a series of biological indicators based on inflammation and/or nutritional status, such as the neutrophil-to-lymphocyte ratio (NLR) and platelet-to-lymphocyte ratio (PLR), have been reported as efficient tumor biomarkers [8–10]. The systemic immune-inflammation index (SII), as a relatively new inflammatory index based on peripheral lymphocyte, neutrophil, and platelet counts, have been reported to have high diagnostic value for the prognosis of cancer [11, 12]. SII has been suggested to be effective in reflecting the inflammatory status and thus may be an underlying biomarker for prognosis prediction. Several studies have reported that the SII is associated with the prognosis of patients with malignancies. However, few studies demonstrated that SII index could have helped in predicting the clinical progression of the patient with COVID-19 [13, 14].

COVID-19 pandemic is a serious cause of increased mortality in patients having malignancy. Therefore, to help predict the clinical progression of those patients with COVID-19 who have multiple comorbidities as cancer, we need various indices whose parameters we can easily measure and rapidly calculate.

Although, as an early warning score, ANDC which is stands for age (A), NLR (N), d-dimer (D) and C-reactive protein (C) has been recently proposed as an early warning score to predict the mortality risk for COVID-19 cases, no malignancy-related data have been issued about ANDC on COVID-19 patients as yet [15].

In this retrospective study, we aimed for an evaluation of SII, ANDC and PNI for their performance in various malignancy cases, and their prognostic value for predicting the mortality due to COVID-19.

## Methodology

We performed a retrospective observational study on 186 patients with SARS-CoV-2, who were diagnosed with COVID-19 by real-time PCR testing and hospitalized due to COVID-19 pneumonia at Prof. Dr. Murat Dilmener Emergency Hospital, pandemic 3-rd level, in Istanbul, from September 01, 2020 to December 31, 2020. Among the patients selected from the hospital electronic database, 75 had various malignancies, and the rest (111) had no malignancy. Both groups were matched with respect to age and comorbidity based on propensity score matching. Patients who had no clinical and laboratory data and who had pneumonia caused by other pathogens were not included in the study. Other criteria for exclusion from the study were to be younger than 18 years old or to have previous hematological disorders such as acute/chronic leukemia, myeloproliferative disorders, acute/chronic liver diseases. None of the patients were admitted to the intensive care unit (ICU). Demographic data and comorbidity were recorded for all patients. On the basis of COVID-19 related examinations, their respiratory rate, oxygen saturation by pulse oximetry (SpO2), and mean oxygen requirement at hospitalization duration were recorded. The hospital has accredited laboratories standardized for internal and external quality assurance measures to monitor the precision and accuracy of the tests performed. In all cases, blood samples were obtained from peripheral vein within 24 h of hospitalization. PNI was calculated as 10 × serum levels of albumin (g/L) + 0.005 × absolute lymphocyte count (/mm3). Also, SII was calculated as the product of the neutrophil and platelet counts divided by the lymphocyte count. In addition to that, recently developed ANDC as an early warning score to predict the mortality risk, was calculated as (1.14 × age − 20) (years) + 1.63 × NLR + 5.00 × D-dimer (mg/L) + 0.14 × CRP (mg/L).

All patients were scanned with spiral computerized tomography (CT) on admission. Radiologist-evaluated CT signs were classified into three categories, mild, moderate and severe involvement. [16]. All the cases enrolled in the study were managed in accordance with the COVID-19 treatment protocol of Turkish Health Ministry [17]. The research was first registered in the data of Turkish Health Ministry Scientific Research Committee and then reviewed and approved by the Local Ethics Committee.

All statistical analyses were performed in commercially available SPSS software v.21 (Statistical Package for the Social Sciences Inc., Chicago, IL, USA). Patient characteristics were summarized using descriptive statistics. Continuous variables were compared using either the unpaired t-test to compare two variables or one-way analysis of variance to compare multiple variables. Categorical variables were compared using the Chi-square test. Mann–Whitney U test was used to evaluate the continuous variables having nonnormal distribution. A p < 0.05 was accepted as statistically significant. ROC analysis was carried out to identify an index for the prediction of mortality rate.

## Results

No significant difference in baseline characteristics and comorbidities were found between the the malignancy (75) and none malignancy (111) groups which were equalized using the propensity score matching. Also, the disease status and radiological involvement were not found to be significantly different between the two groups (Table 1). The respiratory rate, the need for oxygen supplement, the levels of blood phosphorus and lactate dehydrogenase were all found to be significantly higher in the malignancy group. On the other hand, the albumin level was found to be significantly lower in malignancy group. There were no significant differences in NLR, MLR, PLR, SII, PNI and ANDC between the two groups. Mortality ratio was found to be significantly higher in malignancy group, even though the duration of hospitalization was found to be statistically similar. The odds ratio for mortality was 2.39 (%95 CI 1.80–3.16) for the patients with various malignancies (Tables 1, 2). The distribution of patients with respect to different malignancies is given in Table 3 with a %15 predominance of lung cancer.

**Table 1:** Demographical and clinical futures of patients

Baseline characteristics	Malignancies (n = 75)	No malignancies (n = 111)	*P* values
Age, y	64.69 ± 12.98	64.81 ± 6.35	NS
Sex, f/m	28/47	41/70	NS
O2 support, L/per min	4.72 ± 7.25	2.36 ± 3.92	= 0.005
SpO2, under oxygen support; median	93.95 ± 2.11	94.26 ± 1.86	NS
Respiratory rate, per minute	21.65 ± 5.44	18.76 ± 3.69	< 0.001
Body temperature, °C	37.0 ± 0.80	36.93 ± 0.66	NS
Systolic blood pressure, mmHg	126.87 ± 20.21	127.95 ± 18.16	NS
Diastolic blood pressure, mmHg	68.64 ± 10.29	70.98 ± 10.02	NS
Heart rate, per minute	85.05 ± 16.47	81.65 ± 12.81	NS
Arterial hypertension on treatment	33 (44%)	50 (45%)	NS
Diabetes mellitus on treatment	20 (26.7%)	21 (18.9%)	NS
Chronic atrial fibrillation	2 (2.7%)	5 (4.5%)	NS
Heart failure	2 (2.7%)	9 (8.1%)	NS
Prior coronary artery disease	8 (10.7%)	24 (21.6%)	NS
Prior stroke	6 (8%)	7 (6.3%)	NS
Chronic obstructive pulmonary disease	7 (9.3%)	5 (4.5%)	NS
Asthma bronchiale	3 (4%)	13 (11.7%)	NS
Chronic kidney disease	4 (5.3%)	6 (5.4%)	NS
CT results (n, %)			NS
Mild involvement	14 (%18.6)	27 (%24.3)	
Moderate involvement	39 (%52)	60 (%54)	
Severe involvement	22 (%29.4)	24 (%21.7)	
Disease Status (n, %)			NS
Moderate	31 (%41.3)	57 (%51.4)	
Severe	44 (%58.7)	54 (%48.6)

**Table 2:** Laboratory parameters, duration of hospitalization and indices of all cases

Laboratory parameters	Malignancies (n = 75)	No malignancies (n = 111)	*P* values
Neutrophil, cells/mL	5.36 ± 3.55	5.26 ± 3.00	NS
Lymphocytes, cells/mL	1.04 ± 0.60	1.15 ± .46	NS
Monocytes, cells/mL	0.53 ± 0.49	0.53 ± 0.25	NS
Platelets, cells/mL	239.03 ± 117.81	240.86 ± 85.37	NS
Hematocrit, %	36.16 ± 5.59	37.32 ± 4.36	NS
Glucose, mg/dL	154.25 ± 91.40	142.54 ± 70.70	NS
Urea, mg/dL	45.68 ± 29.45	40.19 ± 24.82	NS
Creatinine, mg/dL	0.96 ± 0.52	1.03 ± 1.26	NS
ALT, U/L	39.90 ± 34.63	41.59 ± 33.98	NS
AST, U/L	42.75 ± 30.04	41.50 ± 23.59	NS
Lactate dehydrogenase, U/L	370.12 ± 215.85	316.56 ± 104.38	= 0.02
Potassium, mEq/L	4.23 ± 0.60	4.17 ± 0.52	NS
Sodium, mEq/L	137.35 ± 3.95	137.43 ± 3.25	NS
Magnesium, mg/dL	1.95 ± 0.32	2.04 ± 0.28	NS
Calcium, mg/dL	8.50 ± 0.79	8.63 ± 0.50	NS
Phosphour, mg/dL	3.29 ± 0.81	3.02 ± 0.72	= 0.01
C-reactive protein, mg/L	107.56 ± 89.77	97.55 ± 70.88	NS
Procalcitonin, ng/mL	1.70 ± 11.72	1.24 ± 7.97	NS
Ferritin, mcg/L	631.45 ± 773.96	516.52 ± 517.85	NS
D-dimer, mg/L	0.81 ± 0.99	0.66 ± 0.77	NS
Fibrinogen, mg/dL	527.91 ± 153.35	522.56 ± 123.76	NS
INR	1.06 ± 0.14	1.05 ± 0.14	NS
Troponin I, ng/mL	23.97 ± 87.29	21.30 ± 104.60	NS
Albumin, g/L	3.36 ± 0.56	3.52 ± 0.47	= 0.04
NLR	7.02 ± 7.17	5.52 ± 4.56	NS
PLR	303.89 ± 287.45	242.56 ± 144.02	NS
MLR	0.16 ± 0.34	0.14 ± 0.15	NS
SII	1733.50 ± 2291.64	1445.58 ± 1658.90	NS
ANDC	83.08 ± 25.87	79.87 ± 17.12	NS
PNI	33.74 ± 5.74	35.25 ± 4.79	NS
Duration of hospitalization, day	11.38 ± 6.08	10.27 ± 5.0	NS
Mortality ratio, n, %	13 (%17.3)	3 (%2.7)	< 0.001

SII, systemic immune-inflammation index; PNI, prognostic nutritional index; ANDC, early warning score; NLR, neutrophil lymphocyte ratio; PLR, platelet lymphocyte ratio; MLR, monocyte lymphocyte ratio

**Table 3:** Distribution of malignancies

Malignancies n = 75			
Lung cancer	15	Colon cancer	7
Head and neck cancer	6	Pancreas cancer	2
Breast cancer	7	Urine bladder cancer	5
Gynecological cancer	4	Lymphoma	6
Prostate cancer	9	Other	14

**Fig. 1:**
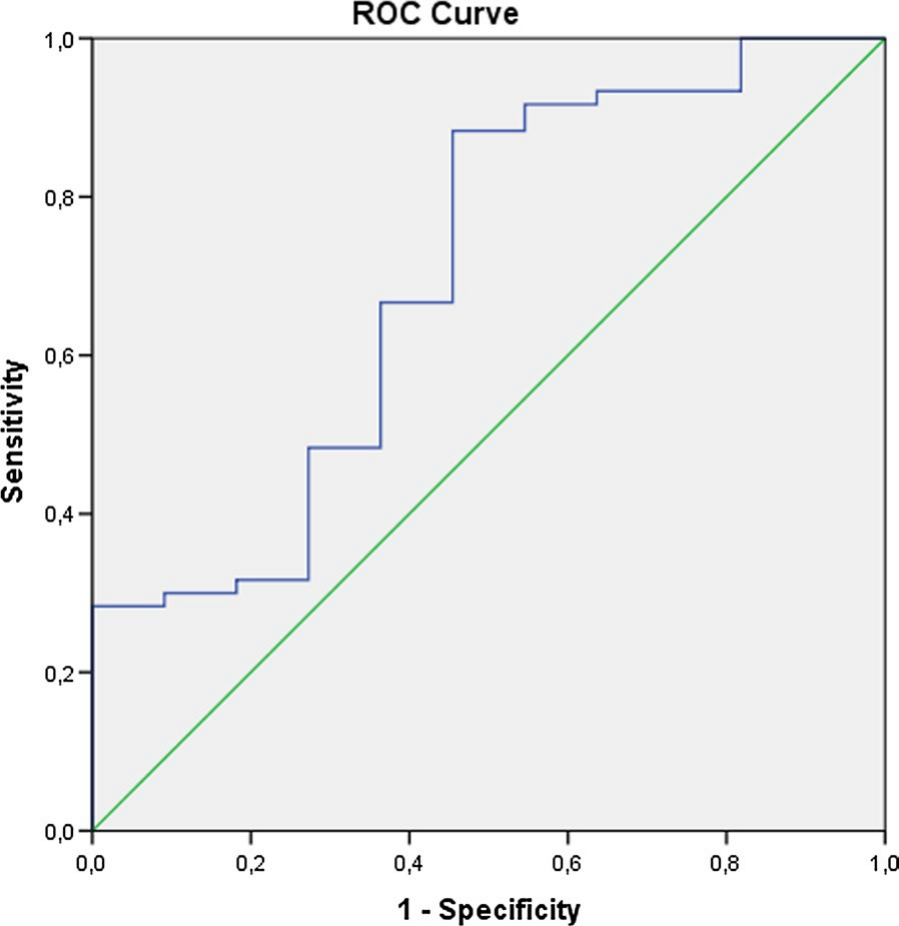
The ROC curves of PNI scoring system for predicting in-hospital mortality. AUC is 0.70 (95% CI 0.52–0.88) at 287 cut-off, with sensitivity and specificity 88% and 55%, respectively

**Fig. 2:**
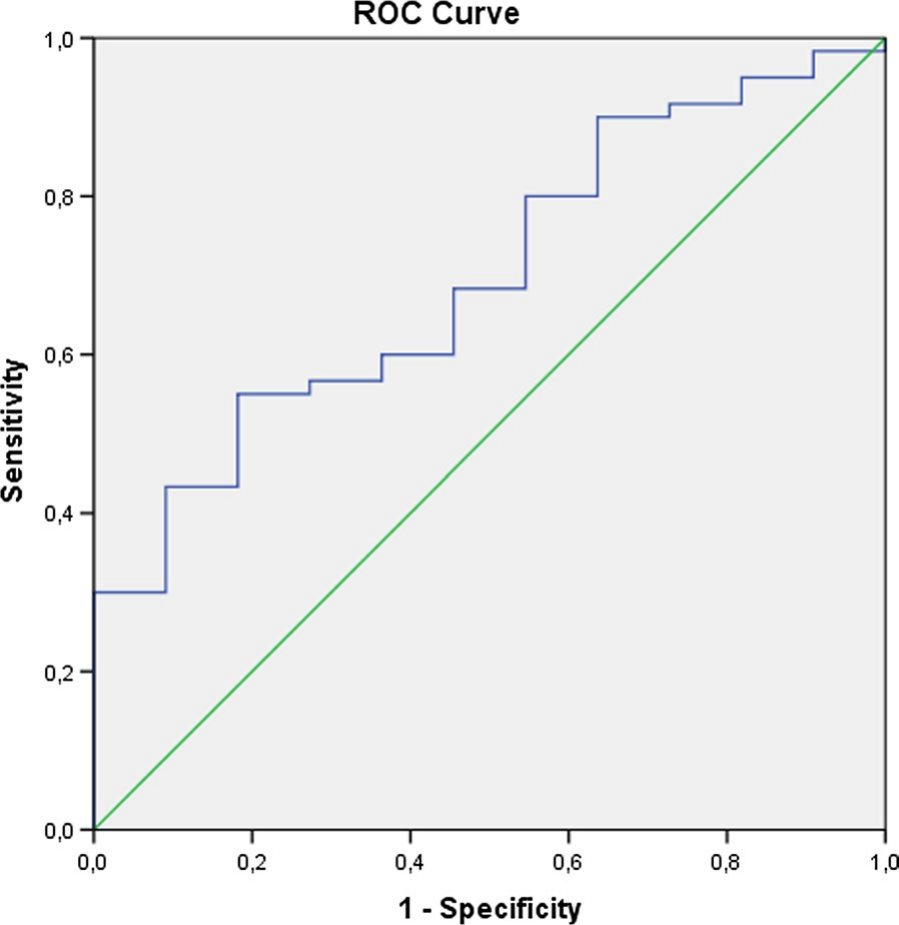
The ROC curves of ANDC scoring system for predicting in-hospital mortality. AUC is 0.69 (95% CI 0.54–0.84), at 100 cut-off, with sensitivity of 80% and specificity of 46%

When the deceased patients were compared with the survivors in the malignancy group, it was found that they yielded a lower PNI in contrast to a higher ANDC, both indices being statistically significant compared in two subgroups. However, there was no difference observed in SII between the deceased patients and survivors (Table 4). Having observed a statistically significant difference between deceased patients and survivors due to their indices (PNI and ANDC), we performed ROC analysis. Regarding the observations made by ROC analyses, the following findings on deceased patients were obtained. The ROC curves of PNI scoring system for predicting in-hospital mortality were depicted in Fig. 1, and its AUC (area under curve) was determined to be 0.70 (95% CI 0.52–0.88) at 287 cut-off, with sensitivity and specificity 88% and 55%, respectively. The ROC curves for the ANDC scoring system was shown in Fig. 2, and its AUC was 0.69 (95% CI 0.54–0.84), at 100 cut-off, with sensitivity of 80% and specificity of 46%.

**Table 4:** Indices of deceased and surviving patients with malignancies

	Deceased patients with malignancies (n = 13)	Surviving patients with malignancies (n = 62)	*P* values
SII	2519.89 ± 2188.53	1589.33 ± 2298.36	NS
PNI	29.86 ± 6.12	34.45 ± 5.43	= 0.01
ANDC	98.25 ± 25.61	80.30 ± 25.15	= 0.03

## Discussion

Considering the high infectivity and mortality rates of COVID-19 with malignancies, early diagnosis of the disease is essentially critical. Blood tests and simple scoring systems play an important role in the early diagnosis of the disease, considering the information they provide to physicians regarding the inflammatory process. Several hematological parameter abnormalities were reported for COVID-19 patients, particularly for those who were severe or critically ill [18–22]. Neutrophils, the most abundant white blood cells in the circulation, are one of the major constituents of the immune system. However, their role in viral infections still remains unclear. In addition, the lymphopenia is observed in many virus-related diseases which causes a cytokine storm, thus resulting in a multi-organ failure and death in severe COVID-19 cases [23]. Recent studies indicate that NLR, MLR and PLR can be used as independent prognostic markers for disease severity in COVID-19 [24, 25]. The SII comprehensively summarizes the balance between the immunity and inflammatory status of the host. It has been already suggested as a prognostic biomarker in sepsis patients [26]. In addition, the SII has also been shown to be associated with a worse survival rate in different cancer types [27–29].

Recently, Fois et al., showed that the SII was the most significant prognostic biomarker for survival in patients with SARS-CoV2 [14]. Their retrospective study, including 119 patients showed that higher SII values were associated with increased mortality rate. Another retrospective study yielded similar results for SII indicating that it was a powerful marker for calling for an invasive ventilator support and preparing for a worse clinical outcome for the COVID-19 patients [13]. In our study, NLR, PLR, MLR and SII did not differ in two groups which were matched according to the age, gender and comorbidities using propensity score matching. Furthermore, SII did not differ in deceased and surviving patients in the malignancy group. These results may lead to a conclusion that COVID-19 may not arouse an adequate inflammatory response in malignancy patients. Therefore, SII as an index defining the instability in the inflammatory response may not be suggested as a prognostic indicator in the follow-up of in COVID-19 patients with malignancies.

Recently, Liang, et al. observed that COVID-19 patients with cancer showed a higher risk and frequency of severity compared with those patients without cancer [3].

Previous studies showed that lower PNI was correlated with a lower survival rate in patients having various malignancies, such as esophageal squamous cancer, colorectal cancer, gastric cancer, and lung cancer [30–32]. Nowadays, PNI is an important biomarker that can be used to discriminate COVID-19 severity, according to the results of several studies [33–37].

In our study, we found no difference in PNI and ANDC between with malignancy and without malignancy groups in COVID-19 patients. This can be partially accounted for the lack of existence of an adequate inflammatory response and a poor nutritional status for malignancy group. Another factor might be a lower level of albumin in no malignancy group which stands out as an acute phase effect in COVID-19 infection. Although the sample size is small, a significant reduction in PNI scores in deceased patients compared with those of the surviving can be interpreted as an indicative score in predicting the mortality.

In current literature, to our knowledge, there is no information available whether PNI and ANDC have any sort of prognostic capability for predicting in-hospital mortality rates of COVID-19 cases with patients having various cancer types. There is a lack of data on PNI and SII for various malignancies indicated by heterogenous type of tumors. Furthermore, ANDC is a recently developed score for predicting the mortality of COVID-19 patients. This is anticipated to be one of the first studies on ANDC in COVID-19 patients having malignancies. Our results indicate that PNI and ANDC differ significantly in deceased and surviving patients in the malignancy group. As it involves the additional consideration of parameters as d-dimer and age, ANDC seems to be a more reliable measure than SII in predicting the mortality in COVID-19 patients. Even though the cancer types were heterogeneous in our cohort, PNI was also observed to have a predictive quality for predicting the mortality.

Finally, phosphorus is known to have an important role in the metabolism of cells, especially during mitosis. It has been previously reported that the growth of cancer in the body is associated with an increased level of phosphorus in the blood in all cancer patients [38]. In addition, serum albumin levels provide useful prognostic significance in cancer [39]. Hypoalbuminemia is a result of combined effects of inflammation and poor nutritional status in patients with chronic disease and cancer [40]. In our study, we found an increased level of blood phosphorus within reference range, which might be associated with cancer in malignancy group. Also lower albumin levels might be associated with acute phase response and/or poor nutrition.

There are several limitations in our study. First, it is a retrospective design. Second, due to the limited number of cases, some of the conclusions are preliminary, especially those on the prospective impact of PNI and ANDC on COVID-19. These results need to be further validated in a larger population with a longer follow-up period.

## Conclusion

Our results show that, as far as COVID-19 cases with malignancies are concerned, PNI and ANDC, both as independent biomarkers, exhibit a certain predictive ability on determining the in-hospital death. On the other hand, SII does not suggest to be a sensitive score as a prognostic tool in COVID-19 cases in the presence of malignancy.

## Data Availability

The authors confirm that the data supporting the findings of this study are available within the article (and/or) its supplementary materials. The data that support the findings of this study are available from the corresponding author, Muge Bilge, upon reasonable request. Correspondence and requests for materials should be addressed to the corresponding author.
